# Surgery for recurrent inflammatory pseudotumor of the lung

**DOI:** 10.1186/1477-7819-9-133

**Published:** 2011-10-18

**Authors:** Taichiro Goto, Kumi Akanabe, Arafumi Maeshima, Ryoichi Kato

**Affiliations:** 1 Department of General Thoracic Surgery, National Hospital Organization Tokyo Medical Center, Tokyo, Japan; 2 Department of Pathology, National Hospital Organization Tokyo Medical Center, Tokyo, Japan

## Abstract

**Background:**

Cases of recurrent inflammatory pseudotumor have only rarely been reported. The treatment for recurrent pseudotumor is surgery. Patients not eligible for surgery require different treatment, and the optimal type of the treatment is controversial.

**Case Presentation:**

A 54-year-old woman was noted to have an abnormal shadow in the right middle lung field on chest X-ray. Computed tomography of the chest revealed an infiltrative lesion in the right segment 4 and a nodule in the right segment 8. She underwent right middle lobectomy and partial resection of the right segment 8. Histopathology revealed non-atypical lymphocytes and plasma cells infiltrates, leading to the diagnosis of the lymphoplasmacytic type of inflammatory pseudotumor. During postoperative follow-up, chest computed tomography revealed a nodular lesion in the left segment 3 and an infiltrative lesion in the right segment 2. Left segment 3 segmentectomy and right segment 2 wedge resection were performed. The histopathological findings were similar to those of the first surgical specimen, leading to the diagnosis of recurrent lymphoplasmacytic type of inflammatory pseudotumor.

**Conclusion:**

Surgical cases of recurrent inflammatory pseudotumor of the lung have been reported only very rarely. We believe that surgery is the best treatment for recurrent inflammatory pseudotumor of the lung when patients are eligible.

## Background

Inflammatory pseudotumor is a rare disease of unknown etiology. The histopathological correlates include a spectrum of fibroblastic or myofibroblastic proliferation with a varying infiltrate of inflammatory cells, including plasma cells, lymphocytes and histiocytes. Inflammatory pseudotumor of the lung is generally treated surgically, and the prognosis of patients undergoing complete surgical resection is favorable [1-7]. However, patients with recurrent inflammatory pseudotumor of the lung have rarely been reported and no effective treatment for such disease has been established. Here, we present a surgically treated case of intrapulmonary recurrence of inflammatory pseudotumor of the lung.

## Case Presentation

**Figure 1:**
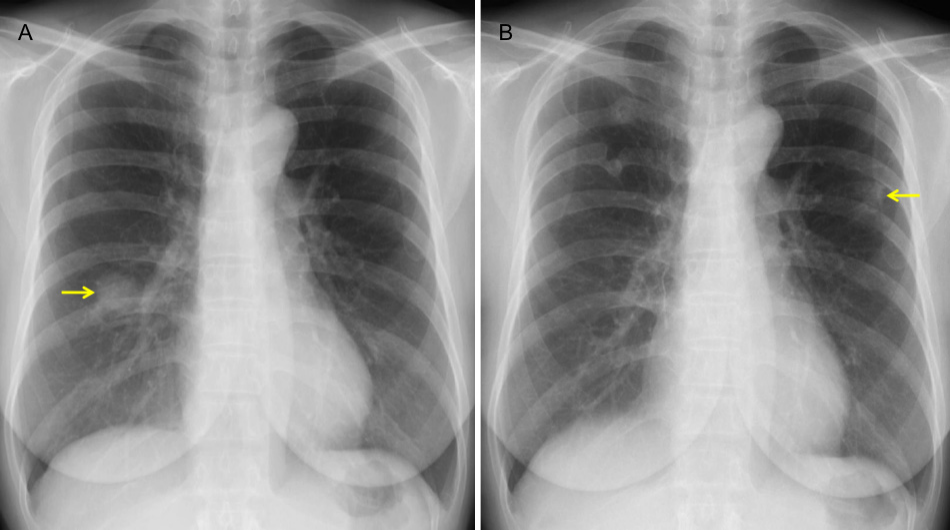
**Chest X-rays taken before the first (A) and second (B) operation**. (A) Chest X-ray shows an ill-defined consolidative lesion in the right middle lung field. (B) Chest X-ray shows an ill-defined consolidative lesion in the left middle lung field. The arrows indicate the lesions.

**Figure 2:**
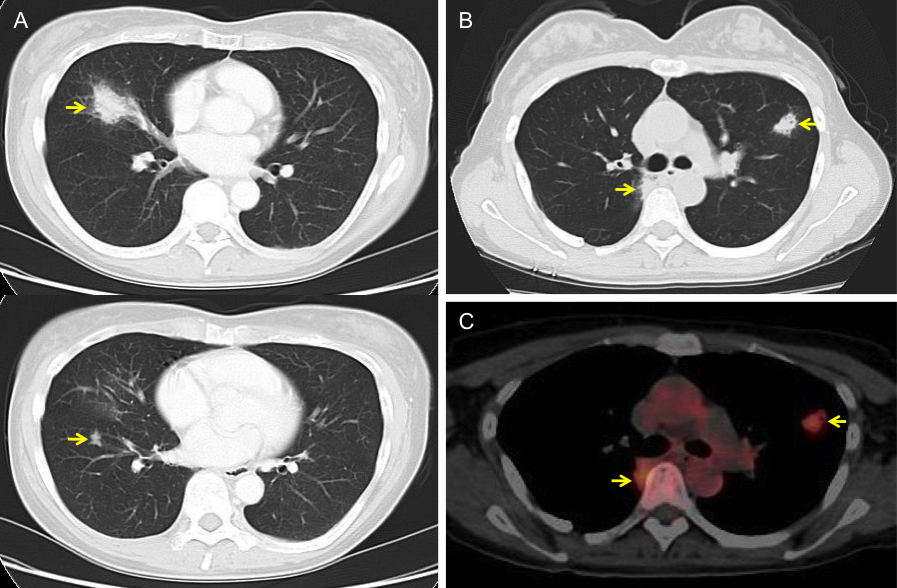
**CT and PET/CT performed before the first (A) and second (B, C) surgery**.  A, A 30-mm infiltrative lesion and a 6-mm nodule are seen in the right S4 and S8, respectively. B, An 18-mm nodular lesion and a 16-mm infiltrative lesion are seen in the left S3 and right S2, respectively. C, PET/CT shows fluorodeoxyglucose uptake within these lesions. The arrows indicate the lesions.

**Figure 3:**
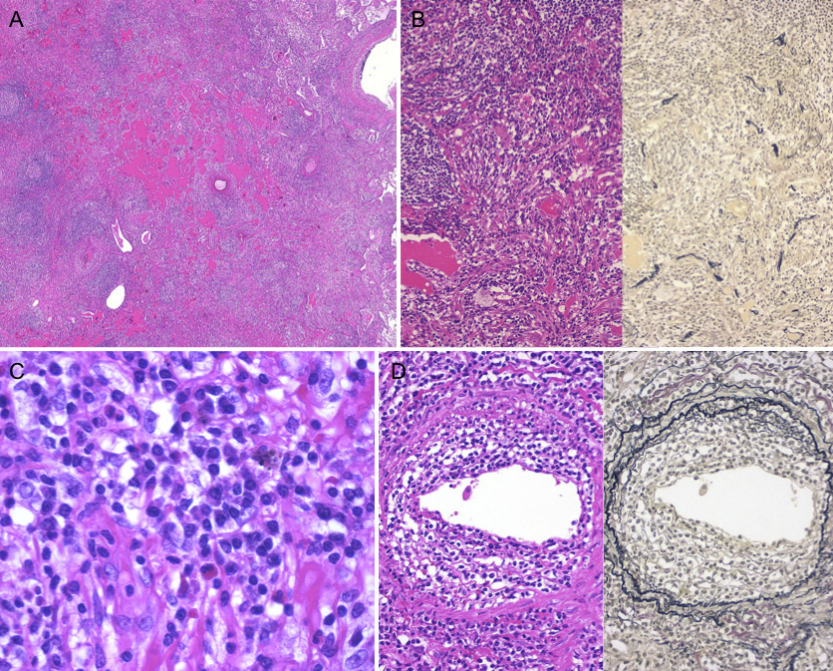
**Histopathology of the first surgical specimen**. A, Lymphoid hyperplasia with germinal centers and exudative changes. B, Proliferation of collagen fibers and destruction of alveolar elastic fibers (left, hematoxylin and eosin staining; right, Elastica-van Gieson staining). C, Infiltrating lymphocytes and plasma cells without atypia. D, Lymphocytic infiltration of vascular walls (left, hematoxylin and eosin staining; right, Elastica-van Gieson staining).

The patient was a 54-year-old woman with a history of 8 pack years of cigarette smoking. Her past medical history was unremarkable. During a medical check-up, a shadow in the right middle lung field was found on chest X-ray (Figure 1A). She was referred to our department for computed tomography (CT) of the chest, which revealed a 30-mm infiltrative lesion in the right segment 4 (S4) and a 6-mm nodule in the right S8 (Figure 2A). Blood samples did not exhibit any abnormalities, including serum immunoglobulin G4 (IgG4), and the tumor markers NSE, CEA, SCC and SLX were within normal limits. As bronchoscopy was nondiagnostic, right middle lobectomy and partial resection of the right S8 were performed. Histopathology showed non-atypical lymphocytes and plasma cells infiltrates, which together had formed lymph follicles with germinal centers (Figure 3A-C). Immunostaining showed that CD20-positive B cells were present in the germinal center, and CD3-positive T cells were observed around them, thus displaying a normal cell distribution. The B cells in the germinal center were positive for CD10, but negative for bcl-2, excluding the possibility of lymphoma. Infiltrates comprised of collagen fibers and damaged alveolar elastic fibers were found (Figure 3B). Lymphocytic infiltration of the vascular walls was present (Figure 3D). There was no preferential immunostaining for immunoglobulin G, immunoglobulin M, immunoglobulin A, the kappa light chain or lambda light chain observed in antibody-producing cells, with no evidence of monoclonal proliferation of B cells or plasma cells. No increase was observed in the number of IgG4-positive plasma cells. Both the S4 and S8 tumor yielded pathological findings similar to the sort described above, and this confirmed the diagnosis of the lymphoplasmacytic type of inflammatory pseudotumor.

**Figure 4:**
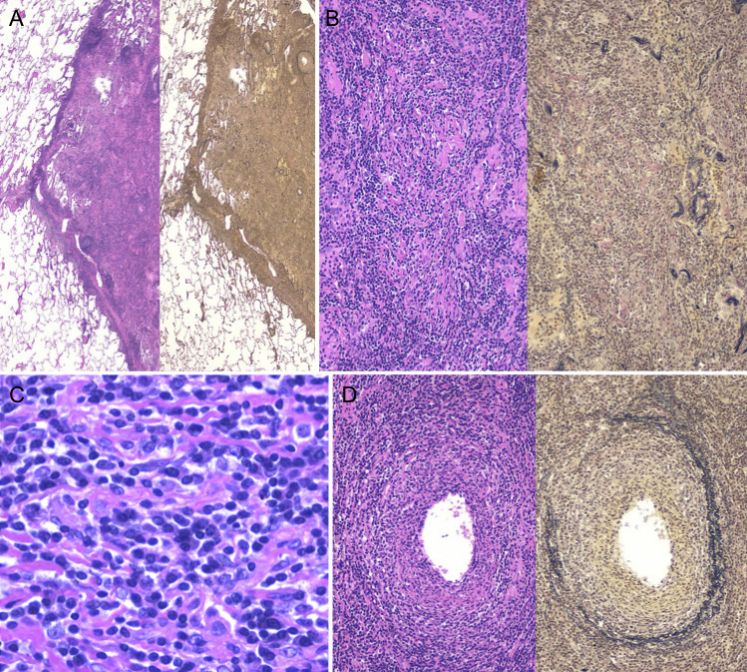
**Histopathology of the second surgical specimen**. A, A nodular lesion well circumscribed by interlobular septa. Lymphoid hyperplasia with germinal centers and exudative changes (left, hematoxylin and eosin staining; right, Elastica-van Gieson staining). B, Proliferation of collagen fibers and destruction of alveolar elastic fibers (left, hematoxylin and eosin staining; right, Elastica-van Gieson staining). C, Infiltrating lymphocytes and plasma cells without atypia. D, Lymphocytic infiltration of vascular walls (left, hematoxylin and eosin staining; right, Elastica-van Gieson staining).

On 4 year postoperative follow-up, chest X-ray revealed a nodular lesion in the left middle lung field (Figure 1B). Chest CT revealed an 18-mm nodular lesion in the left S3 and a 16-mm infiltrative lesion in the right S2 (Figure 2B). Bronchoscopy was nondiagnotic once again. Positron emission tomography (PET)/CT showed maximum standardized uptake values of 2.7 and 3.2 in the masses in the left S3 and right S2, respectively (Figure 2C). These were high fluorodeoxyglucose uptake lesions with maximum standardized uptake values ≥2.5, and, considering the shape of the tumors, either primary lung cancer or recurrence of the inflammatory pseudotumor was suspected. It was critically important to differentiate inflammatory pseudotumor from lung cancer, and, even when a determination of recurrent inflammatory pseudotumors was made, resection was indicated in an effort to achieve long-term survival. Therefore, left S3 segmentectomy was performed at first. The histopathology findings were similar to those of the surgical specimens obtained in the first operation (Figure 4A-D), therefore recurrence of the lymphoplasmacytic type of inflammatory pseudotumor was diagnosed. At this point, the residual right S2 lesion was also considered to be a recurrent inflammatory pseudotumor, but the patient did not wish to receive non-surgical treatment. Since the patient planned to move immediately before surgery, video-assisted thoracoscopic wedge resection of right S2 was performed at another hospital, and the same type of inflammatory pseudotumor was diagnosed histopathologically. Currently, 18 months after the last lung surgery, the patient remains free of disease.

## Conclusions

Inflammatory pseudotumor is comprised of a variable mix of inflammatory and mesenchymal cells, including plasma cells, histiocytes, lymphocytes and fibroblasts. Inflammatory pseudotumor usually arises within the lung, although it may also occur at many other sites [8]. Most of the reported cases were in patients younger than 40 years of age, with a mean age of 27 - 50 years [9]. Both genders are equally affected. No special geographic or ethnic predominance has been reported. The incidence of this lesion in the lung is between 0.04 and 1.0% in the general population [10-13]. Whether an inflammatory pseudotumor is a reactive lesion or a spontaneous neoplasm is controversial, and the precise etiology at present is still unknown. An IgG4-related immunopathological process has attracted attention as a cause of inflammatory pseudotumor in recent years [14]; however, in this patient, the serum IgG4 level was in the normal range, and the IgG4-positive plasma cells in the tumor were immunohistochemically few in number, excluding any association with IgG4-related sclerosing disease. Approximately half of these patients do not exhibit clinical symptoms, and the lesions are often detected incidentally on chest radiographs, while 26-56% of patients have been reported to show clinical symptoms, such as cough, hemosputum, dyspnea and chest pain [13].

The radiological features of inflammatory pseudotumors of the lung have been analyzed by Agrons et al. [9]. Chest CT reportedly reveals a single nodule or mass in approximately 90% of these patients, with multiple nodules in 5% [9]. Secondary infiltration of the hilum, mediastinum and airways is apparent in 16% of patients [9]. PET/CT is usually positive, as in the present case [15]. It is often difficult to make the diagnosis of inflammatory pseudotumor on preoperative imaging findings alone, as there is a highly complex differential diagnosis. Histology specimen obtained by bronchoscopic biopsy or transthoracic fine-needle aspiration are often equivocal. Hence, surgery, such as video-assisted thoracic resection or open lung biopsy, is often required [10,16].

Matsubara et al. categorized three types of inflammatory pseudotumor based on the predominant cell types and main histological characteristics [13]: (i) an organizing pneumonia type with gradual resolution of intra-alveolar exudates; (ii) a fibrous histiocytoma type with storiform proliferation of plasmacyte and lymphocyte aggregations; and (iii) a lymphoplasmacytic type with aggregation of both plasmacytes and lymphocytes, as in the case reported here.

The treatment of choice for pulmonary inflammatory pseudotumor is surgery [1-7]. Inflammatory pseudotumors of the lung usually do not recur after complete resection [3,5]. The prognosis of patients who undergo radical resection is excellent [1-7]. Nevertheless, relapse can occur even many years after resection [6,17], and disease-related deaths have been reported [6,10,18]. In a comparably large series of 23 surgically treated cases over 47 years, only three recurrences were reported, all due to incomplete resection [10]. Two have had subsequent complete excision of enlarged residual tumors with no evidence of recurrence 8 and 9 years later [10]. To our knowledge, this paper is the first report on the surgical resection of metachronous lesions in different parts of the lung, rather than residual tumor growth.

According to Cerfolio et al., patients with recurrent disease who are suitable for surgery should undergo reresection [10]. Their long-term follow-up documented that complete resection leads to extended disease-free intervals. In our case, left S3 segmentectomy and right S2 wedge resection were performed for the purposes of both diagnosis and treatment. We selected surgery because (i) it was necessary to differentiate inflammatory pseudotumor from lung cancer, (ii) even if it was a recurrent inflammatory pseudotumor, there were only two lesions in the lung, and their resection was expected to afford long-term survival, and (iii) the patient did not wish to receive prolonged non-surgical treatment. It is reported that radiotherapy and steroids have each been employed for anatomically and functionally inoperable patients, and also in patients with recurrent disease [19-22]. However, the results of those treatments are widely variable, ranging from ineffective to complete regression [10,20,22]. A larger number of long-term follow-up cases will be needed to establish a clear treatment algorithm.

## Consent

Written informed consent was obtained from the patient for the publication of this case presentation and accompanying images. A copy of the written consent is available for review by the Editor-in-Chief of this journal.

## Abbreviations

S: segment; CT: computed tomography; IgG4: immunoglobulin G4; PET: positron emission tomography.

## Competing interests

The authors declare that they have no competing interests.

## Authors' contributions

TG wrote the manuscript. TG, KA, and RK performed surgery. AM carried out the pathological examination. RK was involved in the final editing. All authors read and approved the final manuscript.
